# Protein Glycation in Plants—An Under-Researched Field with Much Still to Discover

**DOI:** 10.3390/ijms21113942

**Published:** 2020-05-30

**Authors:** Naila Rabbani, Maryam Al-Motawa, Paul J. Thornalley

**Affiliations:** 1 Department of Basic Medical Science, College of Medicine, QU Health, Qatar University, Doha P.O. Box 2713, Qatar; 2 Diabetes Research Center, Qatar Biomedical Research Institute, Hamad Bin Khalifa University, Qatar Foundation, Doha P.O. Box 34110, Qatar; malmotawa@hbku.edu.qa; 3 College of Health and Life Sciences, Hamad Bin Khalifa University, Qatar Foundation, Doha P.O. Box 34110, Qatar

**Keywords:** glycation, advanced glycation end products (AGEs), methylglyoxal, glyoxalase, dicarbonyl stress, unfolded protein response, *Arabidopsis*, *Brassica*, crops

## Abstract

Recent research has identified glycation as a non-enzymatic post-translational modification of proteins in plants with a potential contributory role to the functional impairment of the plant proteome. Reducing sugars with a free aldehyde or ketone group such as glucose, fructose and galactose react with the N-terminal and lysine side chain amino groups of proteins. A common early-stage glycation adduct formed from glucose is N_ε_-fructosyl-lysine (FL). Saccharide-derived reactive dicarbonyls are arginine residue-directed glycating agents, forming advanced glycation endproducts (AGEs). A dominant dicarbonyl is methylglyoxal—formed mainly by the trace-level degradation of triosephosphates, including through the Calvin cycle of photosynthesis. Methylglyoxal forms the major quantitative AGE, hydroimidazolone MG-H1. Glucose and methylglyoxal concentrations in plants change with the developmental stage, senescence, light and dark cycles and also likely biotic and abiotic stresses. Proteomics analysis indicates that there is an enrichment of the amino acid residue targets of glycation, arginine and lysine residues, in predicted functional sites of the plant proteome, suggesting the susceptibility of proteins to functional inactivation by glycation. In this review, we give a brief introduction to glycation, glycating agents and glycation adducts in plants. We consider dicarbonyl stress, the functional vulnerability of the plant proteome to arginine-directed glycation and the likely role of methylglyoxal-mediated glycation in the activation of the unfolded protein response in plants. The latter is linked to the recent suggestion of protein glycation in sugar signaling in plant metabolism. The overexpression of glyoxalase 1, which suppresses glycation by methylglyoxal and glyoxal, produced plants resistant to high salinity, drought, extreme temperature and other stresses. Further research to decrease protein glycation in plants may lead to improved plant growth and assist the breeding of plant varieties resistant to environmental stress and senescence—including plants of commercial ornamental and crop cultivation value.

## 1. Protein Glycation in Plants: Three Papers Setting the Scene

In 2009, we published the first report on the steady-state levels of protein oxidation, nitration and glycation adducts in cytosolic protein extracts from leaves of *Arabidopsis thaliana* (thale cress) [1]. The cytosolic protein contents of the early-stage glycation adduct, N_ε_-fructosyl-lysine (FL), and eight advanced glycation endproducts (AGEs) were presented. This highlighted an aspect of the proteome of higher plants that is intuitive but had been little investigated: proteins are glycated in plants. In 2014, Takagi et al. showed that methylglyoxal (MG)—a precursor of the major quantitative AGE, hydroimidazolone MG-H1—is formed during photosynthesis in chloroplasts isolated from leaves of spinach [2]. In 2017, Bilova et al. published a proteomics study identifying proteins modified by AGEs in *Arabidopsis thaliana* [3]. These studies revealed that higher plants produce glycating agents as a part of their vital photosynthetic metabolism, and the proteome of plants is continually subjected to glycation forming early glycation adducts and AGEs [4]. Further research in this area is, therefore, important to improve understanding of plant growth, resistance to environmental stress and senescence—including for varieties of commercial importance for ornamental and crop production. Below, in this review, we give a brief introduction to glycation, glycating agents and glycation adducts, and, following recent developments in mammalian glycation, we consider dicarbonyl stress, the susceptibility of the plant proteome to functional inactivation by arginine-directed glycation and the role of glycation in the activation of the unfolded protein response (UPR) in plants [5,6]. The latter is likely linked to the recent suggestion of the involvement of protein glycation in sugar signaling in plant metabolism [7]. We limit our coverage to higher plants.

## 2. Glycation: The Maillard Reaction

Protein glycation is the non-enzymatic reaction of simple reducing sugars and related saccharide derivatives with proteins in a complex series of sequential and parallel pathways called the Maillard reaction [8]. Reducing sugars have an aldehyde or ketone group by which reactions occur with the protein substrate, typically on N-terminal and lysine side chain amino groups. In higher plants, examples of simple reducing sugars involved in protein glycation are glucose, fructose and galactose. Glucose reacts with the N-terminal amino groups and lysine residue side chain amino groups of proteins to form an initial Schiff’s base, which undergoes an Amadori rearrangement to form fructosamines: N_α_-1-deoxyfructosyl N-terminal amino groups and N_ε_-fructosyl-lysine (FL). The reactivity of reducing sugars with N-terminal amino groups is usually faster than that with lysine side chain amino groups because of the lower pK_a_ of the former, but lysine side chains are present at higher concentrations than N-termini in the plant proteome. Some lysine side chain amino groups may also be activated towards glycation by interaction with neighboring cationic lysine and arginine residues, decreasing the pK_a_ of the lysine sidechain amino group target [9]. Sucrose, a non-reducing sugar, is the main vehicle for sugar transport in plants. This fact greatly decreases the risk of glycation of the plant proteome by sugar in transit in the plant body (roots, stems and leaves) (44).

Common saccharide derivatives studied in glycation reactions are phosphorylated glycolytic intermediates, such as glucose-6-phosphate (G6P) and ribose-5-phosphate (R5P)—the latter an intermediate of the pentosephosphate pathway and Calvin cycle of photosynthesis in plants [10]. They react with and modify the N-terminal and lysine side chain amino groups of proteins. There are also reactive dicarbonyl saccharide derivatives—such as glyoxal, MG and 3-deoxyglucosone (3-DG) [11]. Dicarbonyl metabolites are arginine-directed glycating agents, forming predominantly hydroimidazolone derivatives such as methylglyoxal-derived hydroimidazolone MG-H1 and related structural isomers, and analogous hydroimidazolones from glyoxal and 3-DG [12,13]. For the reaction of glyoxal with arginine, the ring-opened rearrangement of the initial dihydroxyimidazolidine to N_ω_-carboxymethyl-arginine (CMA) is favored [14]. Glyoxal reacts with lysine residues to form the AGE N_ε_-carboxymethyl-lysine (CML). CML is mainly formed by the oxidative degradation of FL [15], with formation also by the glycation of lysine residues by ascorbic acid [16]. Glyoxal is formed in lipid peroxidation and the slow oxidative degradation of monosaccharides and proteins glycated by glucose [11]. MG is mainly formed by the trace-level degradation of triosephosphates, glyceraldehyde-3-phosphate (GA3P) and dihydroxyacetonephosphate (DHAP) [17]. GA3P is an intermediate in photosynthesis, and GA3P and DHAP are intermediates in glycolysis, gluconeogenesis and glyceroneogenesis in lipid synthesis. 3-DG is formed by the enzymatic and non-enzymatic degradation of proteins glycated by glucose [11,18].

Glycating agents and glycation adduct residues in proteins have been detected in plants. Glycation has been in studied in plants of both research interest and commercial ornamental and crop relevance [1,3,19,20,21,22,23,24]. The levels of glycating agents and glycation adduct residues in proteins vary with light and dark cycles, the stage of development and environmental stresses [1,24]. The molecular structures, metabolic source and likely functional significance of glycation adducts in plants are summarized in Table 1.

There are also enzymes of anti-glycation defense that suppress protein glycation in plant tissues: the glyoxalase system, which metabolizes glyoxal and MG and thereby suppresses the formation of related AGEs [25]; aldoketo reductases (AKRs) that also metabolize glyoxal, MG and likely 3-DG [26]; and ribulosamine/erythrulosamine 3-kinase—a putative protein-repair enzyme that deglycates proteins glycated by R5P [10]. There is also an acylamino acid-releasing enzyme that degrades glycated proteins [27].

Glycation adducts in plant proteins are formed by a slow in situ rate of protein glycation. They are removed by the degradation of glycated proteins by cellular proteolysis. Some glycation adducts are repaired by deglycation enzymes [10,27] and also by the slow spontaneous reversal of the protein glycation, where glycation adducts have moderate stability [12,13,28]. The levels of glycation adducts measured, therefore, are the steady-state levels maintained by these conflicting processes. The steady-state levels of protein glycation adducts are influenced by light, the stage of development, the season, environmental stresses, nutrients and other factors influential for plant metabolism [1] (Table 2). Protein glycation has been implicated in the deterioration of plant seeds in storage [29]. The presence of enzymes in plants that suppress protein glycation and repair glycated proteins [25,30]—the enzymatic defense system against glycation [31]—suggests that glycation of the plant proteome poses a threat to plant physiology and growth.

## 3. Glycation in *Arabidopsis thaliana*

Studies of protein glycation in *Arabidopsis thaliana* under normal growth and stress conditions are summarized in Table 2. Protein glycation was studied in *Arabidopsis thaliana* by assaying protein glycation adducts in cytosolic protein extracts, using the reference method of liquid chromatography-tandem mass spectrometry (LC-MS/MS). This involves the prior exhaustive enzymatic hydrolysis of proteins and quantitation of glycation adducts, glycated amino acids, by stable isotopic dilution analysis LC-MS/MS [13,39,40]. Under basal conditions, the mean extent of protein modification by major glycation adducts was: FL residues, 26%; MG-H1 residues, 4%; and CML residues, 3% [1].

There was a relatively high content of FL residues in plant protein on entering the daylight period—ca. 3 mmol/mol lys. This glycation adduct increased during and beyond the daylight period, when photosynthesis leads to a 10-fold increase in the glucose concentration of the leaf tissue [41] (Table 2). The increase in FL residue content reflects this increased exposure to glucose. The CML residue content of plant protein was relatively high, 0.35–0.71 mmol/mol lys, and showed a similar trend to the change in the FL residue content. This may be due to the high content of FL residues and ascorbic acid [42]—precursors of CML residue formation [15,16]. Excess light stress also increased the CML and G-H1 residue content of total leaf protein by ca. 2-fold. CML and G-H1 adduct residues are formed by the glycation of proteins with glyoxal, which may relate to periods of increased lipid peroxidation and formation of glyoxal [32].

MG-derived MG-H1 residues were the AGE of highest content in the *Arabidopsis thaliana* proteome at ca. 2 mmol/mol arg [1]. Other MG-derived AGE residue contents, N_ε_-(1-carboxyethyl)lysine (CEL) and methylglyoxal-derived lysine dimer MOLD, were ca. 10-fold and 400-fold lower than MG-H1. All the AGE residues, except for CML, tended to show oscillatory diurnal behavior, where the maxima of residue contents occurred in the middle of the light and dark periods and lower levels of AGE residue contents occurred between these times [1].

In recent studies, the boronate affinity enrichment method was used to identify proteins in *Arabidopsis thaliana* modified by FL residues, with protein identification by high mass resolution Orbitrap mass spectrometry proteomics. One hundred and twelve glycated proteins were identified [22]. FL modification and the location of the glycation sites in retained proteins were detected through mass spectrometric detection and the fragmentation of peptides in tryptic digests using high sensitivity mass proteomics, with a mass increment of lysine residues (+162 Da). In this approach, however, there is typically a mean peptide sequence coverage of ca. 25%, and, therefore, some glycation adducts are missed [43]. CML, CMA, G-H1 and MG-H1 residues were also detected in the retained proteins—although these adducts do not bind to boronate affinity columns [12,13,44]. A core group of 112 proteins were identified as glycated, including chloroplast ATP synthase (β-subunit) and phosphoglycerate kinase; 90% of the glycated proteins were of chloroplast origin [22]. The abundances of most of these glycated proteins were similar in experiments investigating the effects of heat, light and drought stresses. The numbers of abundance changes of glycated proteins in stress conditions were: light stress, 2; heat stress, 1; diurnal variation, 8; and drought conditions, 17 [22] (Table 2).

Proteins modified by early-stage glycation adducts and AGEs were examined in a proteomics study. AGE residues were detected at 96 different sites in 71 proteins, with age-dependent changes. Unique age-related proteins modified by AGEs (AGE, sequence location) in 9- and 12-week-old plants and pathways involved were: β-carbonic anhydrase-2, chloroplastic (CMA, R202) and ACT domain containing protein ACR9 (MG-H1, R395)—involved in amino acid metabolism; putative fucosyl transferase-7 (CML, K393 and K394)—involved in cell wall biosynthesis; tetratricopeptide repeat-like superfamily protein (CEL, R83; MG-H1, R86)—involved in the oxidative stress response; and CEL in two uncharacterized proteins. Homology modeling revealed glutamyl and aspartyl residues in close proximity (less than 0.5 nm) to these sites in three aging-specific and eight differentially glycated proteins, four of which were modified in catalytic domains [3]. The protein domains of plant proteins susceptible to glycation have not been widely studied. In mammalian proteins, the domains susceptible to glycation by MG were the tailless complex polypeptide-1 (TCP-1) and GroEL protein domains of chaperonins, the 14-3-3 domain, the α/β subunits of the proteasome, class I and class II aminoacyl-tRNA synthetases, actin and Rossmann-like α/β/α sandwich fold [6]. Similar domains in plant proteins may be susceptible to glycation by MG.

A comprehensive study of the changes in proteins modified by AGEs of *Arabidopsis thaliana* in osmotic stress was reported [19]. Plants were grown from seeds and at 6 weeks and then transferred to new growth medium with and without polyethylenglycol-8000 to provide drought stress and the accumulation of osmolytes, amino acids and carbohydrates. After the application of this osmotic stress for 3 days, changes in 31 stress-specific and 12 differentially AGE-modified proteins reflecting AGEs at 56 different sites were found [19]. Monosaccharide autoxidation [45] was proposed as the main stress-related glycation mechanism, and glyoxal, as the major glycation agent in plants subjected to drought [19].

## 4. Dicarbonyl Stress in Plants

Dicarbonyl stress is the abnormal accumulation of reactive dicarbonyl metabolites leading to increased protein glycation and is linked to cell and tissue dysfunction, aging and disease [5]. MG is a key dicarbonyl metabolite contributing to dicarbonyl stress in plants, unavoidably formed by the non-enzymatic degradation of triosephosphates. It is formed at relatively high flux compared to other dicarbonyl metabolites, ca. 0.1% of glucose metabolism, and has high reactivity with proteins [5]. It is precursor of the major AGE in plant proteins quantitatively, MG-H1 [24,46].

We determined the content of glyoxal, MG and 3-DG of leaves of *Brassica oleracea* plants by the reference analytical method of stable isotopic dilution analysis LC-MS/MS [24]. We studied the leaf dicarbonyl content at three stages of development (days post-seeding): cotyledons (6 days), first fully developed mature leaves (30 days) and mature plants (65 days). The glyoxal content of cotyledons was ca. 0.4 nmol/g fresh weight (ca. 0.4 µM), and it was similar at 30 days but increased 2-fold at 65 days. The MG content of cotyledons was ca. 3 nmol/g fresh weight (ca. 3 µM), and it was similar at 30 days but increased ca. 33% at 65 days. The 3-DG content of cotyledons was ca. 8 nmol/g fresh weight (ca. 0.8 µM); it increased to ca. 2-fold at 30 days and then decreased to levels similar to those at the cotyledon stage at 65 days (Table 3). The markedly higher estimates of MG published previously of ca. 50 nmol/g fresh weight [47] were overestimates likelydue to triosephosphate degradation to MG during pre-analytical processing [48]. The mathematical metabolic modeling of in situ glycation in physiological tissues predicts the steady-state cellular concentrations of MG as 1–4 µM [24] The reactivity towards protein glycation with respect to glucose of glyoxal, MG and 3-DG is ca. 5000, 20,000 and 200 times higher, respectively, so MG is expected to be the major dicarbonyl glycating agent in the leaves of *Brassica oleracea*. The mature 65 day *Brassica* plant appears to have been suffering dicarbonyl stress. The increase and later decrease in 3-DG content may reflect the mobilization of glucose metabolism, declining in the later stages of maturity; cf. measurements of glucose and fructose with plant development [49]. The siRNA silencing of Glo1 and accumulation of MG in other species produced an accelerated aging phenotype, whereas the overexpression of Glo1 increased longevity and produced resistance to metabolic dysfunction in aging [50,51]. Therefore, increased MG glycation may impair plant proteome integrity and vitality in older plants and provide a cue for senescence.

The prolonged use of ammonium salts as the sole nitrogen source to plants may result in physiological and morphological disorders leading to decreased plant growth. This is a worldwide problem, constraining crop production. It is common example of abiotic stress [52]. The effect of this on dicarbonyl stress in *Arabidopsis thaliana* was investigated. Changes in the activities of glycolytic enzymes increased the formation and concentration of MG. The excessive accumulation of MG, dicarbonyl stress, produced increased MG-derived AGEs. Dicarbonyl stress may contribute to ammonium toxicity symptoms in *Arabidopsis thaliana* and the ammonium salt impairment of crop plant growth [38].

## 5. Enzymatic Defense Against Glycation—The Glyoxalase System and Aldoketo Reductases

**Figure 1:**
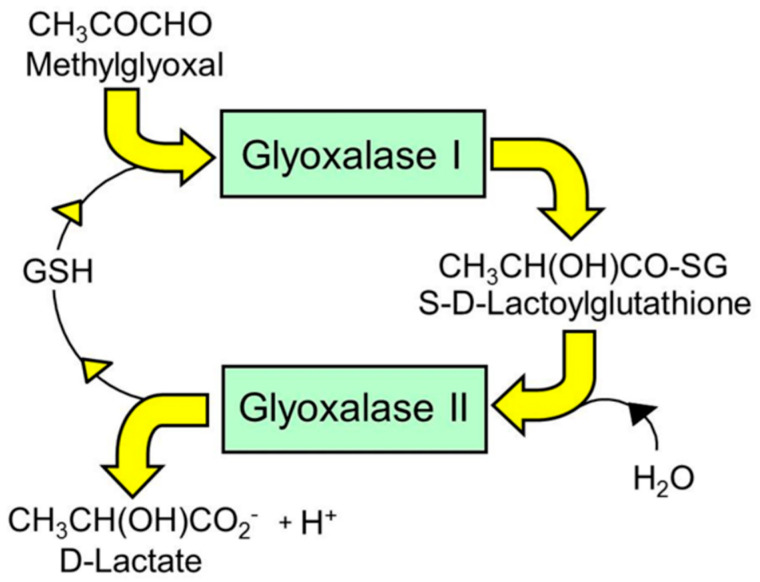
The glyoxalase system. Shown is the metabolism of methylglyoxal to D-lactate. Glyoxal is metabolized similarly to glycolate.

The glyoxalase pathway catalyzes the conversion of MG to D-lactate. It is a two-step, enzymatic pathway: glyoxalase 1 (Glo1) catalyzes the conversion of the hemithioacetal formed non-enzymatically from MG and reduced glutathione (GSH) to S-D-lactoylglutathione (SLG); and glyoxalase 2 (Glo2) catalyzes the hydrolysis of SLG to D-lactate, reforming the GSH consumed in the Glo1-catalyzed step [53,54,55] (Figure 1). There is a further protein called glyoxalase-3 (Glo3) [56], but concern remains on its functional attribution as a glyoxalase involved in MG metabolism physiologically due to its low catalytic efficiency. Catalytic efficiency is defined by the specificity constant k_cat_/K_M_ [57]. The k_cat_/K_M_ values for *Arabidopsis thaliana* Glo1 (isoform 2, accounting for >99% of Glo1 activity) and Glo3 are 1.1 × 10^10^ min^−1^M^−1^ and 3.4 × 10^6^ min^−1^M^−1^, respectively [56,58]; ca. 3100-fold higher for Glo1 than Glo3. The proteomic abundances of Glo1 and Glo3 in *Arabidopsis thaliana* were 1140 and 300 ppm, respectively [59], ca. 4-fold higher for Glo1 than Glo3. The substrate of Glo1 is the hemithioacetal (HA) adduct of MG with reduced glutathione (GSH), whereas the substrate of Glo3 is MG; the concentration of GSH in *Arabidopsis thaliana* is ca. 0.4 mM [60], and the equilibrium constant for HA formation is 333 M^−1^ [61], leading to an HA/MG concentration ratio of 0.17. Taking these factors into account (3100 × 4 × 0.17), the ratio of the rate of metabolism of MG in situ by Glo1/Glo3 is ca. 2100, or only ca. 0.05% of MG is metabolized by Glo3. Therefore, Glo3 does not contribute significantly to MG metabolism in plants under physiological conditions and may have a different, as yet unidentified, function. There is also a protein called glyoxalase-4 with no kinetic characteristics reported, which appears to have a role in the metabolism of high concentrations of exogenous MG (10 mM) [62]. This physiological relevance remains to be evaluated with the markedly lower levels of MG levels found in plants—see Table 3.

The presence of the glyoxalase system in plants has been known for many years [25], where it was initially linked to plant cell growth [63]. Later, it emerged that the overexpression of Glo1 and Glo2 in tobacco plants provided increased tolerance to high exogenous MG and high salinity stress [30]. Genomic analysis identified 19 potential Glo1 and four Glo2 proteins in rice and 22 Glo1 and nine Glo2 proteins in *Arabidopsis thaliana*. The expression profiles differed in response to abiotic stresses in different tissues and during various stages of vegetative and reproductive development [64]. A more recent study identified 40% Glo1 activity and 10% Glo2 activity in the chloroplasts of spinach [65]. In *Arabidopsis thaliana*, under high CO_2_ concentrations, where photosynthesis and the formation of MG are increased, Glo1 and Glo2 expression and activities were increased. This identifies the function of the glyoxalase system in plants as one fundamental to plant biochemistry, providing protection against endogenous dicarbonyl stress [65]. The increased expression of Glo1 was involved in the adaptive response of wild type *Arabidopsis thaliana* and the ascorbate-deficient mutant *vtc2-2* to prolonged exposure to high light intensity [66], now seen as due to protecting against risk of photosynthesis-linked dicarbonyl stress.

There are AKRs in higher plants [67]. These were investigated in *Oryza sativa* (Asian rice) for their role in anti-glycation defense. AKR isoform-1 of rice (OsAKR1) was induced by abscisic acid and various stress treatments, whereas two other AKR genes were moderately stress-inducible. OsAKR1 is an NAPDH-dependent reductase with catalytic activity towards MG and malondialdehyde, the latter formed in lipid peroxidation. The heterologous expression of OsAKR1 in transgenic tobacco plants produced increased tolerance to oxidative stress generated by methylviologen and improved resistance to high temperature. Transgenic tobacco plants also exhibited higher AKR activity and accumulated less MG in their leaves than the wild type plants, both in the presence and absence of heat stress. These results suggest OsAKR1 may also have a role in cytoprotection against dicarbonyl stress in plants [26].

## 6. Glycation in Plants—Considerations for Crops and Other Commercial Aspects

*Brassica oleracea* is an economically and nutritionally important species of plant that is the product of domestication with limited genetic diversity compared to its wild ancestral relatives. The species exists in several different cultivated forms—cabbage, cauliflower, broccoli and Brussels sprouts—and also in its wild form distributed along the European Atlantic seaboard and throughout the Mediterranean area. The reduced genetic base of domesticated *B. oleracea* makes it difficult to find new variants that contribute towards phenotypes capable of resisting stress that are needed to respond to the local and global challenges of food security [68].

Self-incompatibility (the rejection of “self”-pollen) is a reproductive barrier preventing inbreeding, to promote outcrossing and hybrid vigor [69]. Glo1 is a stigma compatibility factor required for pollination to occur and is targeted by the self-incompatibility system. Decreased Glo1 expression reduced compatibility, and the overexpression of Glo1 in self-incompatible *Brassica napus* stigmas resulted in the partial breakdown of the self-incompatibility response, suggesting MG-modified proteins may produce a response leading to pollen rejection [70]. A copy number increase of the GLO1 gene has been explored in *Brassica* plants [71]. In future, crop varieties having functional increased copy numbers of GLO1 may be assessed for improved cross-breeding and improved growth and rigor in maturity and at harvest.

Recombinant human proteins may be expressed in plant-based systems. Examples include human serum albumin and protein immunogenic epitopes for vaccine development and production [72,73]. Recombinant human serum albumins (HSAs) were produced in *Oryza sativa*. LC-MS analysis identified a greater number of hexose-glycated arginine and lysine residues on HSA produced in *Oryza sativa*. There was supplier-to-supplier and lot-to-lot variability in the degree of glycation. Glycation influenced the presence of oligomeric species and tertiary structure. This may have further implications for the use of HSA as a therapeutic product in *Oryza sativa* [72]. The relevance of this glycation is not yet clear but if it impairs the function of HSA—such as ligand binding and esterase activity [74]. It may bring into doubt the use of plant-produced albumin for clinical applications.

## 7. Why Is Glycation Potentially Damaging to Plants?

Glycation in plant proteins is found at relatively low levels, estimated at 26 mol% for FL residues and 4 mol% for MG-H1 residues [1]. Glycation is particularly damaging if it occurs on amino acids in the functional domains of proteins and if modification produces the loss or change of the charge of the target amino acid [75]. To assess the probability of glycation sites being in functional domains in the plant proteome, we applied sequence-based receptor binding domain (RBD) analysis [76] to the proteome of *Arabidopsis thaliana*. In optimized format, RBD analysis involves a plot of the mean hydrophobicity against the mean dipole moment of a window of five amino acid residues moved sequentially along the sequence of a protein (with a gyration angle between two consecutive residues in the sequence of 100° assumed). This approach had 80% accuracy when validated against a database of known interacting proteins [76]. The outcome of the application of RBD analysis to the proteome of *Arabidopsis thaliana* is given herein for the first time in Table 4. The prediction of the proportion of amino acid residues of the RBD region or functional domain suggests that the amino acid residue targets of glycation, lysine and arginine residues, are enriched in functional domains—2.1-fold and 3.7-fold, respectively. By contrast, the amino acid targets of oxidative damage are depleted in functional domains: enrichment—cys, 0.8; met, 0.7; tyr, 0.8; and trp, 0.6. The plant proteome thereby is predicted to be relatively resistant to oxidative functional impairment [77,78]. The glycation of lysine by glucose to form FL residues leads to the retention of sidechain charge, whereas the glycation of arginine by MG produces MG-H1 residues and a loss of charge. Since arginine residues are often in functional domains for salt bridge electrostatic interaction with other proteins, enzyme substrates and nucleic acids, the formation of MG-H1 often produces protein inactivation and misfolding [6,75]. The plant proteome is, therefore, susceptible to glycation by MG at functional domains. This is likely an important physiological source of misfolded proteins and substrates for the UPR in plants [79].

## 8. Role of Dicarbonyl Stress in the Unfolded Protein Response in Plants

Recent studies in mammalian cells suggest that the MG modification of proteins produces misfolding and activation of the UPR [6]. There is an analogous UPR system in plants. It is activated in response to cold, drought, heavy metals and light stress. It preserves proteostasis and protects against the inhibition of photosynthesis [79]. The UPR in plants remains to be fully characterized [6,80]. As in mammalian systems, the inhibitor of enzymatic glycosylation, tunicamycin, has often been used to activate the UPR in the endoplasmic reticulum (ER) or induce ER stress. The physiological activators of unfolded proteins are different, often linked to spontaneous modification such as oxidative damage and MG-derived AGE formation. The latter is particularly damaging to protein structure because it produces a loss of charge, modifications are often in folded and highly structured functional domains, and MG modification also inactivates chaperonins, which catalyze the correct folding of proteins [6]. The link between dicarbonyl stress and the unfolded protein response in plants now deserves investigation.

## 9. Conclusions

**Figure 2:**
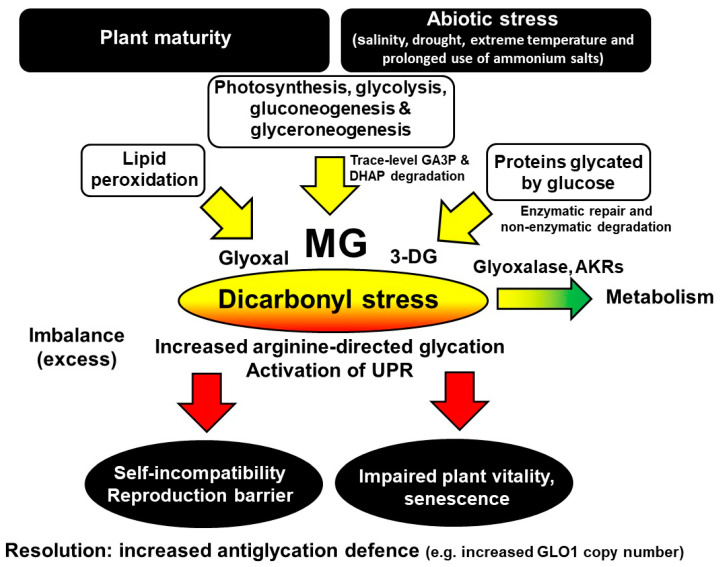
Metabolic drivers, pathophysiological effects and a strategy for the resolution of dicarbonyl stress in plants.

Protein glycation is an unavoidable part of plant metabolism and proteotoxicity, contributing to the damaging effects of excess light, environmental and other stresses in plants. Glycation by glucose and MG produces major early-stage glycation adducts and AGEs, respectively. The levels of these glycating agents and related glycation adducts change with the developmental stage, senescence, light and dark cycles and also biotic and abiotic stresses. Proteomics analysis suggests the susceptibility of the plant proteome to functional inactivation by glycation—particularly glycation on arginine residues by MG. Dicarbonyl stress is an abnormal metabolic state, developing in mature plants during normal growth and cultivation. It may be linked to plant self-incompatibility, impaired plant vitality, pre-mature senescence and sensitivity to abiotic stress—including salinity, drought, extreme temperature and the prolonged use of ammonium salts. Metabolically, dicarbonyl stress is a driver of the increased formation of misfolded proteins and activation of the UPR. Crop breeding for increased functional GLO1 gene copy numbers in plants may produce varieties resistant to dicarbonyl stress, abiotic stresses and senescence with improved breeding and growth characteristics—including plants of commercial ornamental and crop cultivation value. Metabolic drivers, dicarbonyl metabolite, and the consequences of and a strategy for the resolution of dicarbonyl stress in plants are summarized in Figure 2.

## Figures and Tables

**Table 1:** Early-stage glycation adducts and advanced glycation endproducts.

**Glycating Agent**	**Comment**
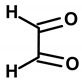 Glyoxal	Formed by the degradation of reducing sugars, glycated proteins, nucleotides and lipid peroxidation [11,32]. Metabolized by the glyoxalase system [5]. Glyoxal is present in solution mainly as mono- and di-hydrates [33].
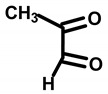 Methylglyoxal (MG)	Formed mainly by the trace-level degradation of GA3P and DHAP [17]. Relatively high flux reactive dicarbonyl. Metabolized by the glyoxalase system [5]. MG is present in solution mainly as mono- and di-hydrates [33]. Precursor of the major AGE, MG-H1.
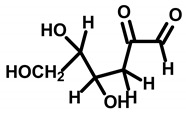 3-Deoxyglucosone (3-DG)	Formed by the degradation of reducing sugars and glycated proteins. Additionally formed by the enzymatic repair of FL [34]. Metabolized by aldoketo reductases [5]. 3-DG is present in solution as a complex mixture of cyclic hemiacetals and hemiketals [33].
**Glycation Adduct**	**Comment**
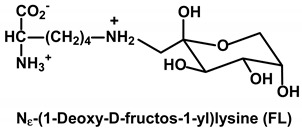	Early-stage glycation adduct [1]. Formed from glucose non-enzymatically and exposure to increased glucose concentration. Repaired intracellularly by fructosamine 3-phosphokinase [35].
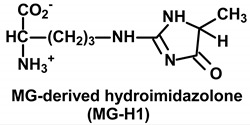	A major quantitative arginine-derived AGE formed from MG. Influenced by the rate of the formation of MG, rate of metabolism of MG by Glo1 of the glyoxalase system and cellular proteolysis. Major AGE in *Arabidopsis thaliana.* Implicated in protein misfolding and, in excess, activation of the UPR.
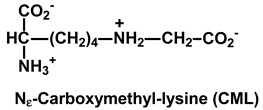	A major quantitative lysine-derived AGE. Formed by the oxidative degradation of FL (major), glycation by glyoxal and by ascorbic acid (usually minor). Increased by light stress in *Arabidopsis thaliana.* The CML/FL ratio is a marker of oxidative stress.
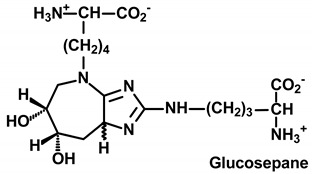	Major quantitative crosslink formed in protein glycation [36]. Produced from the degradation of FL residues with a proximate arginine residue. Content in plant proteins is unknown.
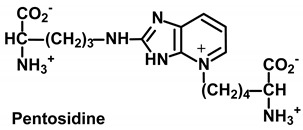	Low-level pentose sugar-derived glycation crosslink and intense fluorophore. Considered to reflect pentosephosphate pathway activity [37].

**Table 2:** Effect of growth conditions on protein glycation in *Arabidopsis thaliana*.

Growth Condition	Effect on Protein Glycation of Glycating	Reference
Daylight to dark growth cycle	Early glycation adduct, FL: 3 mmol/mol lys (daylight entry), increasing to 10 mmol/mol lys (dark entry).	[1]
Diurnal period, heat, light and drought	Glycation adducts detected: CML, CMA, FL, G-H1 and MG-H1. Protein targets: a core group of 112 proteins, including chloroplast ATP synthase (β-subunit) and phosphoglycerate kinase. Glycated protein abundances were similar in heat, light and drought stresses. Glycated proteins with altered abundance were: light stress—2 (RPI3 and TPI, decreased); heat stress—1 (TPI, decreased); diurnal variation—8 (ASP5, FTSH2 and RAN3, increased; AOC2, BAS1, CORI3, OASB, PRK, PRXQ and PURA, decreased); and drought stress 17 (A2, GSA2 and P83484, increased; CAT2, CICDH, CTIMC, CYP18-4, FBP, GGAT1, GLU1, LOX2, P25697, PER34, RBCS-1A, RBCS-3B, TGG2 and TL29, decreased).	[22]
Excess light stress	AGEs increased: G-H1 (0.14 to 0.35 mmol/mol arg) and CML (0.77 to 1.65 mmol/mol lys).	[1]
Osmotic stress	Major glycation adducts detected: CML, CMA and G-H1; 785 glycation sites detected on 724 proteins—33 and 62 glycation sites were unique for control and osmotically stressed plants, respectively. Abundance changes of AGE-modified proteins under osmotic stress (range—2-fold decrease to 27-fold increase): 12 proteins involved in lipid metabolism, DNA supercoils and methylation; protein ubiquitination and degradation; energy metabolism; cell organization and development; cell wall formation; and the regulation of transcription and stress.	[19]
Ammonium NH_4_^+^ salts	MG-H1 and CEL-modified proteins detected by immunoblotting and immunoassays; 15% increase in CEL in ammonium NH_4_^+^-grown plants compared with those in nitrate NO_3_^−^-grown control plants.	[38]

**Table 3:** Reactive dicarbonyl glycating agents in *Brassica oleracea* during development.

Days Post-Sowing	Plant Appearance	Dicarbonyl Metabolite (nmol/g Fresh Weight; Mean ± SD, n = 6)		
		Glyoxal	MG	3-DG
6	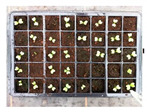	0.38 ± 0.04	2.90 ± 0.81	0.76 ± 0.29
30	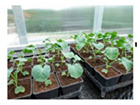	0.46 ± 0.12	3.47 ± 1.21	1.80 ± 1.05 *
65	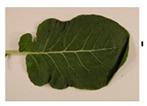	0.81 ± 0.32 **^,OO^	4.08 ± 0.27 *	0.49 ± 0.23 ^O^

*B. oleracea* leaves were from broccoli cv. GDDH33, a well characterized doubled haploid breeding line derived from cv. Green Duke, was sown into F2 compost. The leaves from six plants were removed and flash frozen in liquid nitrogen and stored at −20 °C until analysis. The dicarbonyl contents in the leaves were determined by stable isotopic dilution analysis LC-MS/MS [24]. Briefly, plant leaf (ca. 10 mg fresh weight) was homogenized in 5 % trichloroacetic acid with 0.3 % azide to inhibit peroxidase. Internal standards ([^13^C_3_]MG, [^13^C_2_]glyoxal and [^13^C_6_]3-DG, 2 pmol) were added, mixed and centrifuged (10,000 g, 10 min, 4 °C). Supernatants were derivatized with 1,2-diaminobenzene and analyzed by LC-MS/MS. Significance: * and **, *P* < 0.05 and *P* < 0.01, with respect to 6 days; and ^O^ and ^OO^, *P* < 0.05 and *P* < 0.01, with to respect 30 days; *Student’s t-test*. Data on MG estimation were published previously [24].

**Table 4:** Receptor binding domain (RBD) analysis of the proteome of *Arabidopsis thaliana.*

Amino Acid	Count				
	Proteome	RBD	% AA in Proteome	% AA in RBD	Fold Enrichment
Ala	463,770	25,941	6.5	3.3	0.5
Arg	380,640	150,922	5.3	19.5	3.7
Asn	317,995	44,745	4.4	5.8	1.3
Asp	384,200	52,528	5.3	6.8	1.3
Cys	130,271	10,915	1.8	1.4	0.8
Gln	250,179	38,180	3.5	4.9	1.4
Glu	474,124	70,661	6.6	9.1	1.4
Gly	473,373	30,225	6.6	3.9	0.6
His	160,243	20,712	2.2	2.7	1.2
Ile	392,264	8682	5.5	1.1	0.2
Leu	697,276	28,075	9.7	3.6	0.4
Lys	449,328	101,031	6.3	13.0	2.1
Met	164,360	11,802	2.3	1.5	0.7
Phe	314,311	8387	4.4	1.1	0.2
Pro	341,009	29,637	4.7	3.8	0.8
Ser	636,209	67,405	8.9	8.7	1.0
Thr	369,142	36,395	5.1	4.7	0.9
Trp	90,588	5539	1.3	0.7	0.6
Tyr	209,664	17,971	2.9	2.3	0.8
Val	487,953	15,139	6.8	2.0	0.3
Total:	7,186,899	774,892	100	100	

Amino acid count and RBD analysis applied to 15,938 reviewed protein sequences from the UniProt Knowledgebase (UniProtKB; www.uniprot.org).

## Abbreviations

AGEs	advanced glycation endproducts;
AKR	aldoketoreductase;
CEL	N_ε_-(1-carboxyethyl)lysine;
CMA	N_ω_-carboxymethylarginine;
CML	N_ε_-carboxymethyl-lysine;
3-DG	3-deoxyglucosone;
DHAP	dihydroxyacetonephosphate;
ER	endoplasmic reticulum;
FL	N_ε_-fructosyl-lysine;
GA3P	glyceraldehyde-3-phosphate;
G-H1	glyoxal-derived hydroimidazolone, N_δ_-(5-hydro-4-imidazolon-2-yl)ornithine;
Glo1	glyoxalase 1;
Glo2	glyoxalase 2;
G6P	glucose-6-phosphate;
GSH	reduced glutathione;
HA	hemithioacetal;
HAS	human serum albumin;
LC-MS/MS	liquid chromatography-tandem mass spectrometry;
MG	methylglyoxal;
MG-H1	methylglyoxal-derived hydroimidazolone,
N_δ_-(5-hydro-5-methyl-4-imidazolon-2-yl)-ornithine;	
MOLD	methylglyoxal-derived lysine dimer,
1,3-di(N^ε^-lysino)-4-methyl-imidazolium;	
OsAKR1	aldoketo reductase isoform-1 of *Oryza sativa*;
RBD	receptor binding domain;
R5P	ribose-5-phosphate;
SLG	S-D-lactoylglutathione;
UPR	unfolded protein response.
